# Tumor-Associated Antigens for Specific Immunotherapy of Prostate Cancer

**DOI:** 10.3390/cancers4010193

**Published:** 2012-02-22

**Authors:** Andrea Kiessling, Rebekka Wehner, Susanne Füssel, Michael Bachmann, Manfred P. Wirth, Marc Schmitz

**Affiliations:** 1 Biologics Safety and Disposition, Preclinical Safety, Translational Sciences, Novartis Institutes for BioMedical Research, Novartis Pharma AG, Werk Klybeck, Klybeckstraße 141, Basel CH-4057, Switzerland; E-Mails: andrea.kiessling@novartis.com (A.K.); 2 Institute of Immunology, Medical Faculty, University of Technology Dresden, Fetscherstraße 74, Dresden 01307, Germany; E-Mails: rebekka.wehner@tu-dresden.de (R.W.); michael.bachmann@tu-dresden.de (M.B.); 3 Department of Urology, Medical Faculty, University of Technology Dresden, Fetscherstraße 74, Dresden 01307, Germany; E-Mails: susanne.fuessel@uniklinikum-dresden.de (S.F.); manfred.wirth@uniklinikum-dresden.de (M.P.W.)

**Keywords:** antibodies, dendritic cells, immunotherapy, prostate cancer, T cells

## Abstract

Prostate cancer (PCa) is the most common noncutaneous cancer diagnosis and the second leading cause of cancer-related deaths among men in the United States. Effective treatment modalities for advanced metastatic PCa are limited. Immunotherapeutic strategies based on T cells and antibodies represent interesting approaches to prevent progression from localized to advanced PCa and to improve survival outcomes for patients with advanced disease. CD8^+^ cytotoxic T lymphocytes (CTLs) efficiently recognize and destroy tumor cells. CD4^+^ T cells augment the antigen-presenting capacity of dendritic cells and promote the expansion of tumor-reactive CTLs. Antibodies mediate their antitumor effects via antibody-dependent cellular cytotoxicity, activation of the complement system, improving the uptake of coated tumor cells by phagocytes, and the functional interference of biological pathways essential for tumor growth. Consequently, several tumor-associated antigens (TAAs) have been identified that represent promising targets for T cell- or antibody-based immunotherapy. These TAAs comprise proteins preferentially expressed in normal and malignant prostate tissues and molecules which are not predominantly restricted to the prostate, but are overexpressed in various tumor entities including PCa. Clinical trials provide evidence that specific immunotherapeutic strategies using such TAAs represent safe and feasible concepts for the induction of immunological and clinical responses in PCa patients. However, further improvement of the current approaches is required which may be achieved by combining T cell- and/or antibody-based strategies with radio-, hormone-, chemo- or antiangiogenic therapy.

## 1. Introduction

Prostate cancer (PCa) represents the most common noncutaneous cancer and the second leading cause of cancer mortality among men in the United States, with an estimated incidence of 240,890 cases and an estimated number of 33,720 deaths in 2011 [1]. Whereas patients with localized PCa are often successfully treated with radical prostatectomy and radiotherapy, effective therapeutic options for patients with metastatic hormone-refractory PCa (HRPC) are limited [2,3]. Although treatment with the chemotherapeutic agent docetaxel plus prednisone or the androgen biosynthesis inhibitor abiraterone acetate plus prednisone resulted in an improvement of survival, a substantial proportion of patients do not benefit or have benefit of limited durability [4,5,6]. Therefore, additional treatment strategies are needed to further improve survival outcomes for patients with advanced PCa.

Specific immunotherapy emerged as a promising treatment modality for advanced PCa [7,8]. T cells and antibodies are powerful components of the specific antitumor immune response. CD8^+^ cytotoxic T cells (CTLs) can efficiently recognize and destroy tumor cells which expose peptides derived from intracellular tumor-associated antigens (TAAs) in complex with human leukocyte antigen (HLA) class I molecules on the surface [9]. CD4^+^ T cells recognizing peptides in the context of HLA class II molecules on the cell surface also play an important role in antitumor immunity [10]. CD4^+^ T cells improve the capacity of dendritic cells (DCs) to induce CTLs by the interaction between CD40 on DCs and CD40 ligand on activated CD4^+^ T cells. Furthermore, CD4^+^ T cells provide help for the maintenance and expansion of CTLs by secreting cytokines, and can eradicate tumor cells directly. Clinical studies focusing on the adoptive transfer of naturally occuring human T cells demonstrated tumor regression in cancer patients [11,12]. Following these promising findings, immunotherapeutic strategies which were based on the generation of genetically modified T cells have been designed [13,14]. Human T cells can be engineered to express T cell receptors or chimeric antigen receptors which recognize TAAs on the cell surface in a non-HLA-restricted manner. Clinical trials revealed that both T cell receptor- or chimeric antigen receptor- engineered T cells can mediate tumor regression in cancer patients [15,16]. 

DCs are professional antigen presenting cells (APCs) which display a unique capacity to induce, sustain and regulate T-cell responses [17,18]. In tumor setting, DCs circulate through the blood and migrate to tumor tissues, where they interact with malignant cells. Immature DCs are particularly efficient in the uptake of tumor-derived material. DC maturation can be induced by tumor-derived molecules such as heat shock proteins and high-mobility-group box 1 protein as well as proinflammatory cytokines produced by various tumor-infiltrating immune cells. During maturation DCs migrate from tumor tissues to T cell-rich areas of secondary lymphoid organs, where they activate tumor-reactive CD8^+^ CTLs and CD4^+^ T cells. Owing to their extraordinary capacity to induce and expand tumor-reactive T cells, DCs emerged as promising candidates for cancer vaccination therapy [19,20].

Monoclonal antibodies (mAbs) recognize target structures on the tumor cell surface and mediate their antitumor effects by various mechanisms of action [21]. They are able to efficiently activate the complement system leading to the recruitment of immune cells and tumor cell lysis. In addition, mAbs mediate antibody-dependent cellular cytotoxicity (ADCC) by stimulating tumor-reactive immune cells. They also enhance the uptake of coated tumor cells by phagocytes resulting in an activation of tumor-reactive CD8^+^ CTLs and CD4^+^ T cells. Furthermore, they can interfere with biological pathways essential for tumor growth. Immunotherapeutic strategies based on the administration of mAbs have been successfully introduced into clinic [22]. More recently, bispecific antibodies directed against a tumor cell surface antigen and an activating receptor on immune effector cells are developed that engage these cells into tumor eradication [23,24]. 

Consequently, much attention has been paid to the identification of TAAs that represent attractive targets for T cell- or antibody-based immunotherapy. 

## 2. Proteins Predominantly Expressed in Prostate Tissues

The group of molecules preferentially expressed in normal and malignant prostate tissues comprises prostate-specific antigen (PSA), prostate-specific membrane antigen (PSMA), prostatic acid phosphatase (PAP), prostate stem cell antigen (PSCA), T cell receptor gamma alternate reading frame protein (TARP), transient receptor potential (trp)-p8 and six-transmembrane epithelial antigen of the prostate 1 (STEAP1).

### 2.1. Prostate-Specific Antigen

PSA, a kallikrein-like serin-protease, is almost exclusively expressed by prostate epithelial cells, can be detected in the majority of PCa tissues, and represents the most widely used serum marker for diagnosis and monitoring of PCa [25,26,27,28]. HLA-A2-restricted PSA-derived peptides were identified by using peptide-pulsed or RNA-transfected APCs to activate tumor-reactive CTLs [29,30,31,32,33]. By applying an oligopeptide, which contains several HLA-A2- and HLA-A3-restricted PSA epitopes, the simultaneous induction of tumor-reactive CTLs has been demonstrated [34]. In addition, two HLA-A24-restricted PSA peptides were reported to generate peptide-specific CTLs from PCa patients [35]. One of these epitopes induced HLA-A*2402-restricted CTLs in HLA-A*2402/K^b^-transgenic mice [36]. Furthermore, immunogenic PSA peptides presented by additional HLA class I or HLA class II molecules have been described [37,38,39]. 

Consequently, much attention has been paid to the optimization of delivery strategies for an active immunotherapy such as the transduction of DCs with an adeno-associated virus-based vector which more effectively stimulated PSA-specific CTLs *in vitro* when compared to protein-pulsed DCs [40]. The capability of PSA to induce specific T cell responses has been successfully demonstrated in mouse models. Arredouani *et al*. [41] showed that HLA-A*0201/human PSA-double transgenic mice with prostate-specific and androgen-dependent expression of the PSA transgene develop augmented CTL responses when castrated prior to immunization with an PSA-expressing vaccinia virus, providing a rationale of combining vaccination strategies with androgen deprivation. This concept is further supported by the mitigation of CD4^+^ T cell tolerance to a prostate-restricted model antigen by androgen ablation indicating that specific immunotherapy of PCa may be more efficacious when administered after androgen ablation [42].

### 2.2. Prostate-Specific Membrane Antigen

The integral membrane glycoprotein PSMA represents a marker for normal prostate cells and can be detected in the majority of prostate tumors, particularly in undifferentiated, metastatic HRPC [43,44]. Although original studies indicated a high tissue-specificity of expression [45], PSMA was also found in other normal tissues such as salivary gland, brain, small intestine, renal tubular epithelium and breast epithelium [46]. However, absolute quantification revealed 100- to 1000-fold lower expression levels in non-prostatic tissues [47]. 

HLA-A2-restricted PSMA-derived peptides were shown to induce antitumoral CTL responses *in vitro* [48,49,50]. In addition, immunogenic HLA-A24-restricted peptides [51,52] and two peptides promiscuous for HLA-A11, HLA-A31 and HLA-A33 [38] have been identified. Furthermore, HLA class II-restricted peptides generated by natural PSMA processing and showing promiscuous binding to different HLA-DR variants were identified *in vitro* which efficiently induce T cell responses in human HLA class II transgenic mice [53,54]. PSMA has been subjected to *in vitro* and *in vivo* studies for optimized antigen capability to stimulate T cell responses. In this context, co-transduction of genes encoding the extracellular domain of PSMA and a costimulatory protein using an adenoviral vector has been proven to effectively activate specific T cell responses *in vitro* and to elicit protective and therapeutic anti-tumor immunity in a murine tumor model [55].

Based on the surface expression on PCa cells, PSMA also represents a promising target molecule for antibody therapy. Evaluation of different anti-PSMA mAbs coupled to ricin A and the bismuth-conjugated mab J591 binding to the extracellular PSMA portion revealed target-specific cytotoxicity against PSMA-expressing PCa cells [56,57]. ^213^Bi-J591, humanized ^90^Y-chelate-J591 and ^131^I-J591 have also been shown to markedly reduce the tumor volume in nude mice bearing LNCaP xenografts [56,58]. The effectively internalized antibody conjugates of J415 and J591 coupled to ^131^I or ^111^In showed preferential accumulation in areas of the viable tumor in xenograft models which is a prerequisite for effective radioimmunotherapy [59]. An antibody conjugate of humanized J591 to the immunotoxin saporin elicited potent and selective antitumor effects on PSMA-expressing PCa cells *in vitro* and *in vivo* [60]. Wolf *et al*. [61] developed an anti-PSMA single chain antibody fragment fused to *Pseudomonas* Exotoxin A with PSMA-specificity and therapeutic efficacy in a mouse xenograft model.

The use of an anti-PSMA x anti-CD3 bispecific diabody to selectively activate PSMA-specific CD8^+^ and CD4^+^ T cells and to recruit them to the tumor site revealed efficient inhibition of tumor growth in a xenograft model [62,63]. Another approach for PSMA-specific targeting is based on engineered T cells expressing chimeric anti-PSMA immunoglobulin-T-cell-receptor constructs which were shown to specifically lyse PSMA-expressing PCa cells and retard tumor growth in a mouse xenograft model [64].

### 2.3. Prostatic Acid Phosphatase

The expression of PAP which represents one of the major proteins secreted by prostate epithelial cells is mainly restricted to benign and malignant prostate tissue [65]. Studies investigating PAP expression in non-prostate tissues revealed low mRNA expression levels especially in placenta, kidney and testis [66]. Immunohistochemical staining confirmed extra-prostatic PAP expression [67]. In addition, it has been shown that PAP expression is high in tumors with Gleason scores of 6 and 7 and decreases with higher Gleason scores [68]. Interestingly, PAP was also found to be expressed in adenocarcinomas of different tissues such as gastric, breast and colon cancer [69].

Naturally generated, HLA-A2-binding, immunogenic peptides were identified [50,70] and resulted in specific tumor rejection *in vivo* [71,72]. Additional HLA-A2-restricted PAP-derived peptides were identified by analyzing pre-existing reactive CD8^+^ T cells in the blood of PCa patients and healthy donors [73]. Furthermore, CTL-inducing PAP-derived peptides fitting to other HLA class I molecules have been defined [38,74]. A peptide originally described in the context of HLA-A3 has been shown to additionally bind to HLA-A2 and HLA-A24 and as being capable to induce tumor-reactive CTLs from PBMCs of either allelic HLA variant [75]. Such promiscuous peptides have significant relevance in immunotherapy due to their broad applicability for a large percentage of the patient population. By immunizing HLA class II variant-transgenic mice with PAP protein and subsequent *in vitro* screening for CD4^+^ T cell reactivity directed to PAP sequence motifs of an overlapping 20-mer peptide library, CD4^+^ T cell epitopes for several HLA-DR allelic variants were identified [76,77]. 

The immunotherapeutical potential of vaccination strategies using PAP as target antigen have been demonstrated in mouse models and clinical trials. In a recent study, a DNA vaccine encoding murine PAP induced PAP-specific CTL responses and effectively suppressed tumor growth in a Transgenic Adenocarcinoma of the Mouse Prostate (TRAMP) model [78]. Moreover, in a recent phase III trial patients with advanced PCa were treated with APCs preexposed *in vitro* to PA2024, a fusion protein consisting of human granulocyte-macrophage colony-stimulating factor and PAP [79]. Patients in the sipuleucel-T treatment group experienced a relative reduction of 22% in the risk of death compared with the placebo group. The median survival was 25.8 months in the sipuleucel-T group and 21.7 months in the placebo group. Based on these findings, the United States Food and Drug Administration recently approved sipuleucel-T for the treatment of asymptomatic or minimally symptomatic, metastatic HRPC.

### 2.4. Prostate Stem Cell Antigen

PSCA is a glycosylphosphatidylinositol-anchored cell surface glycoprotein that is expressed in basal and secretory epithelial cells of the prostate and was originally identified by its upregulation in a human PCa xenograft model [80,81]. On transcript level, it is predominantly expressed in the prostate at low mRNA levels detected in placenta, kidney and small intestine [66]. Low protein expression was found in bladder, placenta, neuroendocrine cells of stomach and kidney [81]. PSCA expression is detectable in more than 80% of primary PCa samples and bone metastases and is increased in both androgen-dependent and -independent prostate tumors when compared to the corresponding normal prostate tissues, particularly in carcinomas of high stages und Gleason scores [80,81]. Significant upregulation was also detected in non-organ confined tumors and seminal vesicle invasion when compared with tumors restricted to the prostate [82] as well as in PCa bone metastases [83]. In addition to PCa, PSCA was subsequently identified as a tumor-associated protein of other tumors including pancreatic adenocarcinoma, renal cell carcinoma and diffuse-type gastric cancer [84].

We and others identified HLA-A2-restricted PSCA peptides capable of generating tumor-reactive CTL responses *in vitro* [85,86,87]. We also detected increased frequencies of CD8^+^ T cells recognizing two of these peptides in the blood of PCa patients [86]. Furthermore, an HLA-A24-presented peptide that effectively stimulated CTLs from PCa patients was found [88]. 

Several studies focusing on the immunotherapeutical potential of PSCA were conducted in the TRAMP mouse model displaying PSCA expression pattern in the spontaneously developing PCa. When vaccinated with a viral vector encoding PSCA after priming with PSCA cDNA, TRAMP mice with prostate intraepithelial neoplasia developed antigen-specific CTL responses and displayed a significantly increased survival rate when compared to the control group [89]. Another study using this mouse model revealed that vaccination with recombinant DNA and modified vaccinia virus Ankara vectors encoding PSCA and STEAP1 inhibits PCa progression [90].

PSCA has also been evaluated as target for antibody-based immunotherapy. Anti-PSCA mAbs conjugated to the toxin maytansinoid were effectively internalized by PCa cells resulting in cytotoxicity and regression of xenografts in mice [91]. Furthermore, an inhibited formation and retarded growth of established xenografts, increased long-term survival and reduced metastasis formation were observed in mice treated with the unconjugated anti-PSCA antibody 1G8 [92]. In another study with this antibody, the mechanisms of action were analyzed *in vitro* and *in vivo* and revealed Fc-independent induction of cell death requiring target cross-linking [93]. Several chimeric and humanized anti-PSCA antibody radioconjugates were shown to specifically target PSCA-positive xenografts and to exhibit antitumor effects *in vivo* [94,95]. Furthermore, we generated bispecific antibody constructs directed against PSCA and CD3 on human T cells that engage these immune effector cells into tumor cell killing [96]. In another approach, we modified T cells by the transduction of chimeric antigen receptors which specifically recognize PSCA. The engineered T cells efficiently lysed PSCA-expressing cells [97].

### 2.5. Prostein

The transmembrane protein prostein is typically expressed in normal and malignant prostate tissues and is potentially involved in PCa cell migration and invasion [98,99,100]. We found maintained or even elevated transcript levels in 87% of the primary tumors compared to autologous non-malignant tissue samples [101]. Prostein expression is higher in organ-confined PCa when compared to non-organ-confined tumors [102].

By *in vitro*-stimulation of CD8^+^ T lymphocytes with peptide-loaded DCs we identified an autochthonously generated HLA-A*0201-presented, prostein-derived peptide which was able to stimulate tumor-reactive CTLs [100]. Immunogenic T cell epitopes presented by HLA-B*5101 and HLA-Cw*0501 were also described [103]. 

### 2.6. T Cell Receptor Gamma Alternate Reading Frame Protein

TARP is derived from a unique androgen-regulated transcript of a portion of the non-rearranged T cell receptor gamma-chain locus [104,105]. In males, TARP is expressed in the mitochondria of PCa cells [106]. In females, TARP can be detected in breast cancer [104].

Several naturally generated HLA-A*0201-restricted TARP-peptides which stimulated PCa and breast cancer cell-reactive CTLs *in vitro* were identified [107,108]. In addition, Kobayashi *et al*. defined two TARP-derived HLA class II-binding peptides which were shown to elicit effective CD4^+^ T cell responses [109].

TARP was subject of evaluation of a novel targeting approach using antibodies specifically binding individual HLA class I-peptide complexes on the surface of tumor cells for diagnostic and therapeutic purposes [110]. In this study, an antibody that mimicked the unique specificity for an HLA-A2-TARP peptide complex was able to inhibit the growth of human breast cancer cells in nude mice when fused to *Pseudomonas* Exotoxin A. 

### 2.7. Trp-p8

The gene *trp-p8* encodes a seven-span transmembrane protein with significant homology to a family of Ca^2+^ channel proteins [111]. Trp-p8 expression is mainly restricted to the prostate and is detected in the majority of prostate tumors [111]. Further analysis revealed overexpression in tumors of early stages and low grades when compared to the corresponding normal prostate tissue [112]. We identified an HLA-A*0201-binding peptide which was able to stimulate tumor-reactive CTLs *in vitro* [112]. 

### 2.8. Six-Transmembrane Epithelial Antigen of the Prostate 1

STEAP1 is a transmembrane protein originally identified by its overexpression in a PCa xenograft model that mimics an advanced disease stage [113]. It is predominantly expressed in prostate epithelium, but was also detected in colon and liver at a significantly lower transcript level [113]. STEAP1 is not only overexpressed in different stages and metastases of PCa, but also in a variety of other tumor types including bladder, colon and ovarian cancer [113,114]. 

Several naturally processed HLA-A2-restricted peptides capable of inducing CTLs *in vitro* and *in vivo* as well as three promiscuous CD4^+^ T cell epitopes have been identified [71,115,116,117,118,119]. 

Some vaccination strategies using recombinant cDNA or viral vectors encoding mouse STEAP1 were successful in the induction of specific T cell responses, reduction of tumor growth and increase of survival in mouse models [90,120,121].

Recent data suggest that STEAP1 additionally represents an attractive target for antibody-based immunotherapy as two STEAP-specific mAbs significantly inhibited the growth of PCa xenografts in mice [114].

## 3. Proteins Overexpressed in Various Tumors Including Prostate Cancer

Several potential target structures for specific immunotherapy are not predominantly restricted to the prostate, but are overexpressed in different tumors of epithelial and/or hematopoietic origin including PCa. This group comprises parathyroid hormone-related protein (PTHrP), human telomerase reverse transcriptase (hTERT), survivin, members of the epidermal growth factor receptor family, N-cadherin, erythropoietin-producing hepatocellular receptor tyrosine kinase class A2 (EphA2) and synovial sarcoma X chromosome break point (SSX) proteins.

### 3.1. Parathyroid Hormone-Related Protein

PTHrP is a factor that binds receptors on osteoblasts and induces bone formation. It is highly overexpressed in PCa and other cancers of epithelial origin including gastric, breast, lung, colon, cervical and renal cancer and is considered to be involved in the development of bone metastases [122,123]. Therefore, it might represent a promising immunotherapeutical target for a wide range of tumors, especially bone metastases. 

Four HLA-A2-restricted PTHrP peptides were shown to elicit tumor-reactive CTLs *in vitro*, and two of them were additionally described to induce antitumoral CTL responses *in vivo* [124,125]. Furthermore, HLA-A24-binding peptides were proven to be immunogenic *in vitro* [126]. 

Due to its role in metastasis formation and bone destruction in malignant diseases, neutralizing anti- PTHrP antibodies may provide an interesting immunotherapeutic tool. Promising results have been obtained in a mouse model where neutralizing antibodies inhibited the formation of osteolytic bone metastases of lung cancer cells [127].

### 3.2. Human Telomerase Reverse Transcriptase

Another potential target for specific immunotherapy is hTERT. This molecule is undetectable in most non-transformed somatic cells but is expressed in more than 85% of human tumors including PCa [128]. Several naturally generated HLA-A*0201-restricted CTL epitopes have been described that efficiently activate peptide-specific and tumor-lysing CTLs *in vitro* and *in vivo* [129,130,131,132]. Furthermore, immunogenic hTERT-derived peptides fitting to other HLA class I molecules have been defined [133,134,135,136,137]. Beyond HLA class I epitopes, several promiscuous, naturally generated HLA class II-binding peptides have been identified [138,139]. 

### 3.3. Survivin

Survivin, an inhibitor of apoptosis and promoter of proliferation, is expressed in many tissues during fetal development whereas expression is almost absent in differentiated healthy adult tissues. However, expression can be detected in cells undergoing self renewal as hematopoietic precursor cells, keratinocytes, lymphocytes, activated endothelial cells and epithelial cells of the uterine cervix [140]. Survivin is highly overexpressed in many human tumors including PCa, and its expression correlates with tumor progression, poor prognosis of tumor disease and drug resistence [141,142]. In PCa, survivin has been identified as a mediator of resistance to anti-androgen therapy [143]. The wide expression in cancer and the almost complete absence in differentiated adult tissues together with the functional role for tumor cell survival make survivin a promising target for T cell-based immunotherapy. 

We and others identified two naturally generated HLA-A*0201-restricted peptides which induced specific CTL responses *in vitro* [144,145]. Furthermore, CD8^+^ T cells reactive against one of the previously defined survivin peptides and a peptide modified at an anchor aminoacid were found in the blood of tumor patients [146]. A number of additional CD8^+^ T cell epitopes restricted to other HLA class I molecules were defined by analyzing the peptide specificity of spontaneous CTL responses in cancer patients [147,148] and *in vitro* stimulation of PBMCs [149]. Recently, different HLA class II-associated peptides have been identified in the association with HLA-DR and -DP molecules [150,151].

In pre-clinical models, immunotherapeutical targeting of survivin has been shown to effectively induce T cell responses and to exert anti-tumor effects for various solid tumors [152,153].

### 3.4. Epidermal Growth Factor Receptor Family (HER-2/neu, EGFR, HER-4)

Several cell surface proteins of the ErbB receptor tyrosine kinase family such as c-erbB-1 (EGFR, HER-1), c-erbB-2 (HER-2/neu) and c-erbB-4 (HER-4) are overexpressed in different tumors including PCa [154] and may represent promising target structures for T-cell or antibody-based immunotherapy. 

The tyrosine kinase receptor HER-2/neu is the target structure for the humanized mAb trastuzumab which has been successfully used for the treatment of breast and gastric cancer [22,155]. Recent studies revealed that Her-2/neu is involved in the progression of PCa to androgen-independent disease, and overexpression in primary PCa is associated with a worse clinical outcome such as earlier recurrence and shorter survival [156,157]. A large panel of HLA class I- and class II-restricted HER-2/neu-derived peptides has been identified [158]. Moreover, active immunotherapy has been proven to be effective in mouse models of different solid tumor types [158]. Monoclonal anti-HER-2/neu antibody therapy has been evaluated in pre-clinical models. Growth of established tumors was significantly inhibited by trastuzumab administration in androgen-dependent xenograft models as well as in a combination treatment with the tyrosine kinase inhibitor gefitinib in a HRPC xenograft model [159,160]. Moreover, designed T cells directed to HER-2/neu efficiently targeted PCa bone marrow metastases in a SCID mouse model [161].

EGFR is overexpressed in a significant percentage of PCa and was found to be involved in PCa progression to androgen independence [162,163]. *In vivo* studies which were based on the administration of the EGFR-specific mAbs cetuximab and panitumumab have shown inhibition of tumor growth in various PCa xenograft models [164,165]. Although EGFR has mainly been studied as target stucture of mAbs, several CTL epitopes have been identified [166,167]. 

Recently, c-erbB-4 also emerged as potential target molecule due to its frequent overexpression in PCa [168]. C-erbB-4 antibodies were able to delay the growth of several PCa *in vitro* and in a mouse xenograft model [169,170]. In addition, c-erbB-4-directed mAb treatment could be improved by concomitant radiation therapy [170].

### 3.5. N-Cadherin

N-cadherin is involved in the interaction between cells and extracellular matrix components. Upregulation of N-cadherin is a characteristic feature of tumor progression in the context of epithelial-mesenchymal transition, thereby promoting cell motility, invasiveness and metastasis formation including pelvic lymph node infiltration and bone metastasis in PCa [171,172]. In PCa, N-cadherin upregulation is also associated with dedifferentiation, androgen-deprivation and transition to androgen-independence [173,174]. Despite its significant expression in several normal tissues including nervous system, vascular endothelium and myocardium, it may provide a target structure for advanced androgen-independent and/or metastatic PCa. Promising results were recently obtained by mAb targeting of castration-resistant PCa which markedly reduced the growth of such xenografts, blocked invasion and metastasis and delayed the progression to androgen resistance [175].

### 3.6. Erythropoietin-Producing Hepatocellular Receptor Tyrosine Kinase Class A2

EphA2 is a cell-membrane bound receptor tyrosine kinase which is expressed in a wide panel of normal tissues [176], but is highly overexpressed in many epithelial tumors including PCa [177]. Therefore, EphA2 may provide a target for active and passive immunotherapy in advanced PCa. Several HLA class I and II-restricted peptides have been identified [178,179], and some peptides showed anti-tumor effects in mouse models when pulsed on DCs [180,181].

The immunotherapeutical potential of mAbs with agonistic activity has been intensively evaluated in pre-clinical *in vivo* models. Such antibodies can induce internalization and degradation of the receptor as demonstrated by an effective down-regulation of EphA2 on the cell surface [182]. In addition, they exhibit potent anti-tumor activity in various xenograft models alone or in combination with chemotherapy as a result of reduced tumor cell proliferation, apoptosis induction and reduced microvascular density [182,183]. Enhancement of immune cell effector function by introducing Fc mutations may even potentiate the anti-tumor effects by increased ADCC [184]. Due to the rapid receptor internalization by agonistic antibody targeting, immunoconjugates with cytotoxins may provide very effective tools for tumor targeting [185,186,187]. 

### 3.7. Synovial Sarcoma X Chromosome Break Point Proteins

SSX proteins represent a superfamily of highly homologous cancer-testis antigens with nuclear localization, restricted expression in HLA class I-deficient testis or ovary germline cells and frequent overexpression in tumors of various histological origins, especially in advanced stage cancer [188,189]. Due to the limited expression in normal HLA class I-expressing cells, SSX proteins are particularly attractive immunotherapeutic targets for T cell-based strategies. In PCa, SSX protein expression was recently described as absent in primary tumors, but present in a significant percentage of metastatic PCa samples [190]. Several HLA class I and II-restricted peptides have been identified, and some of them respresent target stuctures for tumors expressing different SSX family members due to their high level of protein homology [188,190,191].

## 4. Conclusions

In recent years, various TAAs have been identified that represent attractive target structures for specific immunotherapy of prostate cancer. These TAAs comprise proteins preferentially expressed in normal and malignant prostate tissues and molecules which are not predominantly restricted to the prostate, but are overexpressed in various tumor entities including PCa. The identification of TAAs and derived HLA class I and class II-restricted T cell epitopes paved the way for the design of novel T cell- or antibody-based immunotherapeutic strategies. CD8^+^ CTLs efficiently recognize and destroy tumor cells. CD4^+^ T cells augment the antigen-presenting capacity of DCs and promote the expansion of tumor-reactive CTLs. Antibodies directed against tumor surface antigens can mediate their antitumor effects by engaging cytotoxic effector cells such as natural killer cells, complement activation, improving the uptake of coated tumor cells by phagocytes, and the functional interference of biological pathways essential for tumor growth. Clinical trials which aimed at the *in vivo*-activation of CD8^+^ CTLs and CD4^+^ T cells by the administration of peptides, proteins, DNA, or TAA-loaded DCs revealed that these approaches were safe and feasible. Furthermore, immunological and clinical responses were induced in PCa patients. Clinical studies including mAbs directed against prostate cancer surface antigens were also conducted and resulted in some clinical responses. However, further improvement of current immunotherapeutic treatment modalities for advanced PCa is required which may be achieved by combining T cell- and/or antibody-based strategies with radio-, hormone-, chemo- or antiangiogenic therapy. 
